# Effects of pre-anthesis low-temperature stress on the mineral components in wheat grains

**DOI:** 10.3389/fpls.2023.1221466

**Published:** 2023-07-27

**Authors:** Wenbin Ji, Xinyi Hu, Meng Kang, Xiaolei Qiu, Bing Liu, Liang Tang, Yan Zhu, Weixing Cao, Leilei Liu

**Affiliations:** ^1^ Key Laboratory for Crop System Analysis and Decision Making, National Engineering and Technology Center for Information Agriculture, Engineering Research Center of Smart Agriculture, Jiangsu Collaborative Innovation Center for Modern Crop Production, Ministry of Education, Ministry of Agriculture, Nanjing Agricultural University, Nanjing, China; ^2^ Jiangsu Key Laboratory for Information Agriculture, Ministry of Agriculture, Nanjing Agricultural University, Nanjing, China

**Keywords:** climate change, fuzzy comprehensive evaluation method, low-temperature stress, mineral components, nutritional quality, wheat

## Abstract

**Introduction:**

The nutritional value of wheat is important to human health. Despite minerals being essential nutrients for the human body, they are often neglected in consideration of the nutritional quality of cereal grains. Extreme low-temperature events have become more frequent due to the current environmental unpredictability, and it is yet unknown how the mineral components in grains are affected by low temperature.

**Methods:**

To provide valuable information for enhancing the nutritional quality of wheat under potential climatic conditions, we treated different cold-sensitive wheat cultivars at four low-temperature levels during the individual and combined stages of jointing and booting in controlled-environment phytotrons.

**Results and Discussion:**

In general, the contents of P, K, Ca, and Zn in the cold-sensitive cultivar (Yangmai16) and K in the cold-tolerant cultivar (Xumai30) were enhanced by low temperature. However, the accumulation of minerals in mature grains was reduced under low-temperature treatment, except for P, Ca, and Zn in Yangmai16. In addition, the mineral content and accumulation in Yangmai16 (except for Fe) were more susceptible to low temperature during the combined stages, while the mineral content and accumulation of K, Fe, and Zn in Xumai30 were more susceptible to low temperature during the booting stage. Moreover, Yangmai16 under extremely low temperatures (T3 and T4) during booting and Xumai30 under all low-temperature treatments during the combined stages had lower comprehensive evaluation values. These findings offer a crucial reference for enhancing the nutritional quality of wheat grains under climate change.

## 1 Introduction

With the development of the social economy and the improvement of living standards, people are paying increasing attention to the role of cereal crop nutritional elements and dietary fiber in human nutrition and health. As essential nutrients for the human body, minerals are a collection of various elements necessary for the formation of tissues and the maintenance of regular physiological functions (Gupta and Gupta, 2014). Typically, humans are unable to produce minerals on their own and must instead obtain them through foods such as cereals and vegetables. Wheat (*Triticum aestivum* L.), one of the world’s major food crops, and the various products from wheat are the primary source of minerals for humans, providing 20−40% of their daily mineral requirements (Zhang et al., 2014; Ma et al., 2021).

In terms of the nutritional quality of wheat grains, protein and starch have long received much scientific attention (Nuttall et al., 2017), but little is known about the mineral components of wheat grains. The mineral components necessary for the normal life processes of plants mainly include phosphorus (P), potassium (K), calcium (Ca), iron (Fe), and zinc (Zn). Ca is a component of the cell wall and the inter-cellular layer (Thor, 2019); when Ca is deficient, the tips or green leaves of plants turn yellow, causing premature senescence of the plants (Shrivastav et al., 2020). P is a component of the plant cytoplasm and nucleus, and plays an important role in plant metabolism (Xu et al., 2022); when P is deficient, the transportation of sugar in plants is hindered, resulting in dwarf plants. K is mainly concentrated in the vigorously growing parts of plants and participates in catalyzing various enzymatic reactions; the lack of K can make plants less resistant to cold or even cause plant death (Ankit et al., 2022). As an important co-factor of some key enzymes, Fe is also an essential element for the synthesis of chlorophyll. Zn and Ca both maintain the stability and integrity of cells; a lack of Zn makes the stem inter-nodes of the plant shorter and causes chlorosis of the leaves (Shrivastav et al., 2020).

The mineral components of wheat grains not only affect the growth and development of wheat but also participate in various biochemical reactions in human cells that are essential for preventing various diseases and maintaining health (Gharibzahedi and Jafari, 2017). Research shows that more than 20 kinds of nutrients necessary to maintain human life and health are mineral nutrients. Based on their proportion in the human body, minerals can typically be divided into two categories: major elements such as Ca, P, and K and trace elements such as Fe and Zn (Welch and Graham, 2004). In the human body, Ca maintains the structure of the skeletal system and participates in physiological metabolic processes such as those related to the cardiovascular, endocrine, and nervous systems (Weaver and Peacock, 2019), while P serves as a substrate for vital physiological processes such as adenosine triphosphate (ATP) synthesis, signal transduction, and bone mineralization; both of these elements are crucial for human health (Serna and Bergwitz, 2020). K helps prevent hypertension and enhances bone health since it maintains normal osmotic pressure and fluid balance in cell function (McLean and Wang, 2021). As the most abundant and critical micro-nutrient in the human body, Fe is involved in several enzymatic activities, oxygen transport, and cellular energy metabolism (Lal, 2020). Nearly all life stages and fundamental bodily processes in humans are affected by Zn activity, including human growth and development, metabolism, and immune function (Hall and King, 2022). However, insufficient intake or excessive consumption of mineral nutrients, especially trace minerals, leads to malnutrition and imbalance, which seriously threaten human health (Lukaski, 2004). Although the rate of global malnutrition has dropped significantly in the past few decades, more than 30% of the world’s population, mainly in underdeveloped areas where plant foods are the staple food, still suffers from ‘hidden hunger’ due to a lack of minerals such as Fe, Zn, and Ca (Beach et al., 2019). Therefore, fortifying mineral nutrition in grains such as wheat is crucial to people’s nutritional health.

Notably, in the context of global climate change, frequent extreme weather events have led to trends of decreasing availability of micro-nutrients (Ca, P, K, Fe, and Zn, among others) in 87 countries with various socioeconomic levels and more severe trends in landlocked developing countries and low-income, food deficient countries (Park et al., 2019), implying that the risk of malnutrition is increasing (Mirzabaev et al., 2023). As an environmental factor necessary for crop growth and development, temperature is closely related to grain quality. The shift of phenological periods to occur earlier in response to climate warming has brought more extreme low-temperature events in the early stage of wheat growth, and approximately 85% of wheat-growing regions in China are at risk of spring cold damage each year, which has serious implications for wheat nutritional quality (Yue et al., 2016). Previous research has suggested that low temperature alters the composition of plant membrane lipids and cell membrane permeability, which in turn directly affects the bio-availability of mineral elements in plants (Lynch and Steponkus, 1987). Gonzalez-Fuentes et al. discovered that large root zone temperature (RZT) fluctuations negatively affected the plant growth, leaf area, and nutrient uptake of strawberries, and low RZT (5°C) resulted in an extremely negative stem water potential, which inhibits plant growth (Gonzalez-Fuentes et al., 2016). Miyasaka et al. studied the effects of different RZTs (constant 8°C, constant 16°C, and a transition from 8°C to 16°C after 23 d) and Ca levels on the mineral content of winter wheat and found that low RZT (8°C) significantly reduced the shoot and root concentration and unit absorption rate for P, S, Cu, Zn, and Mn and that an increase in Ca level enhanced the absorption and accumulation of P and Cu in plants (Miyasaka and Grunes, 1997). In addition, low temperature influences the distribution and overall enrichment levels of mineral components among plant organs mainly by inhibiting the uptake of various mineral nutrients by roots and hindering the above ground transport of nutrients (Lukatkin et al., 2012). Currently, due to climate warming, most studies on wheat quality have focused on the effects of high temperatures, drought, and the combination of elevated CO_2_ concentrations and warming (Nuttall et al., 2017; Zahra et al., 2023). There are fewer studies on the effects of low-temperature stress on grain protein, starch, and amino acids. To better understand the effects of low temperature on wheat grain quality, Labuschagne et al. (2009) exposed bread, biscuit, and durum wheat to an extremely low-temperature environment and found that low temperature significantly reduced sodium dodecyl sulfate (SDS) sedimentation in all varieties. Moreover, temperature-controlled experiments carried out in phytotrons showed that low temperature had a positive effect on the protein concentrations and processing quality of wheat but a negative effect on the starch concentrations and quality of appearance (Liu et al., 2019). Recently, Hu et al. (2022) reported that after low-temperature treatment, leucine (Leu) and threonine (Thr) in wheat grains were near those of the standard protein, while phenylalanine (Phe) and tyrosine (Tyr) were in surplus, and the other essential amino acids did not meet the standard. However, the effects of low temperature on the mineral component content in wheat grains and comprehensive evaluations of plant-specific mineral components in response to low temperature have received little attention.

To examine the effects of low temperature on the mineral nutrients in wheat grains, two wheat cultivars with different cold sensitivities were exposed to different low-temperature levels in controlled-environment phytotrons during the pre-anthesis stage. The objectives of this study were to (1) investigate the response of mineral nutrients in wheat grains to different low-temperature conditions and (2) comprehensively evaluate the effects of low-temperature stress on grain mineral nutrient quality based on fuzzy set theory. These results can provide valuable guidance for enhancing and optimizing the mineral nutritional quality of wheat grains in the context of changing climatic conditions.

## 2 Materials and methods

### 2.1 Data sources

The experiments were conducted during the wheat growing season from 2017 to 2019 in four independent controlled-environment phytotrons at the experimental station of the National Engineering and Technology Center for Information Agriculture (NETCIA), which is in Rugao City (120.33°E, 32.23°N), Jiangsu Province, China. Two winter wheat cultivars with different cold tolerances, Yangmai16 (cold-sensitive) and Xumai30 (moderately cold-tolerant), were planted in plastic pots (30 cm deep and 25 cm in diameter, with five evenly distributed 1 cm drainage holes in the bottom of the pot) with soil that was entirely taken from the field. The dates for sowing in 2017−2018 and 2018−2019 were October 26 and November 1, respectively. Twenty-five seeds were sown evenly in each pot at a depth of 2 cm, and 10 plants were retained in each pot at the three-leaf stage. Other cultivation management measures, such as irrigation, weeding, and pest and disease control, were carried out according to the local optimal management program to prevent other stresses.

To explore the effects of low-temperature stress on the mineral composition quality of wheat grains, three low-temperature periods before flowering were demarcated during the wheat growth and development: the jointing stage (Zadoks 31), the booting stage (Zadoks 45), and both the jointing and booting stages. Four independent controlled-environment phytotrons were used for the low-temperature treatments with different temperature regimes: T1 (6°C/16°C, control treatment), T2 (0°C/10°C), T3 (−2°C/8°C), and T4 (−4°C/6°C), and each low-temperature treatment lasted for 3 days, a design that determined on the basis of Xiao et al. (2018). The wheat plants grown in pots were placed in an ambient environment before and after low-temperature treatment. When the wheat plants reached the corresponding growth period (jointing or booting stage), they were moved into the phytotrons for different low-temperature treatments, and the pots were placed randomly and rotated daily to minimize position effects. The specific treatment details are shown in **Table 1**.

**Table 1:** Summary of the low-temperature stress treatments.

Growing season	Cultivar	Treatment stage	Duration(days)	Start and end dates of treatment(m/dd)
2017−2018	Yangmai16	Jointing	3	3/06−3/08
		Booting	3	3/27−3/29
		Jointing and booting	3 and 3	3/06−3/08 and 3/27−3/29
	Xumai30	Jointing	3	3/18−3/20
		Booting	3	4/08−4/10
		Jointing and booting	3 and 3	3/18−3/20 and 4/08−4/10
2018−2019	Yangmai16	Jointing	3	3/06−3/08
		Booting	3	3/29−3/31
		Jointing and booting	3 and 3	3/06−3/08 and 3/29−3/31
	Xumai30	Jointing	3	3/18−3/20
		Booting	3	4/08−4/10
		Jointing and booting	3 and 3	3/18−3/20 and 4/08−4/10

**Figure 1:**
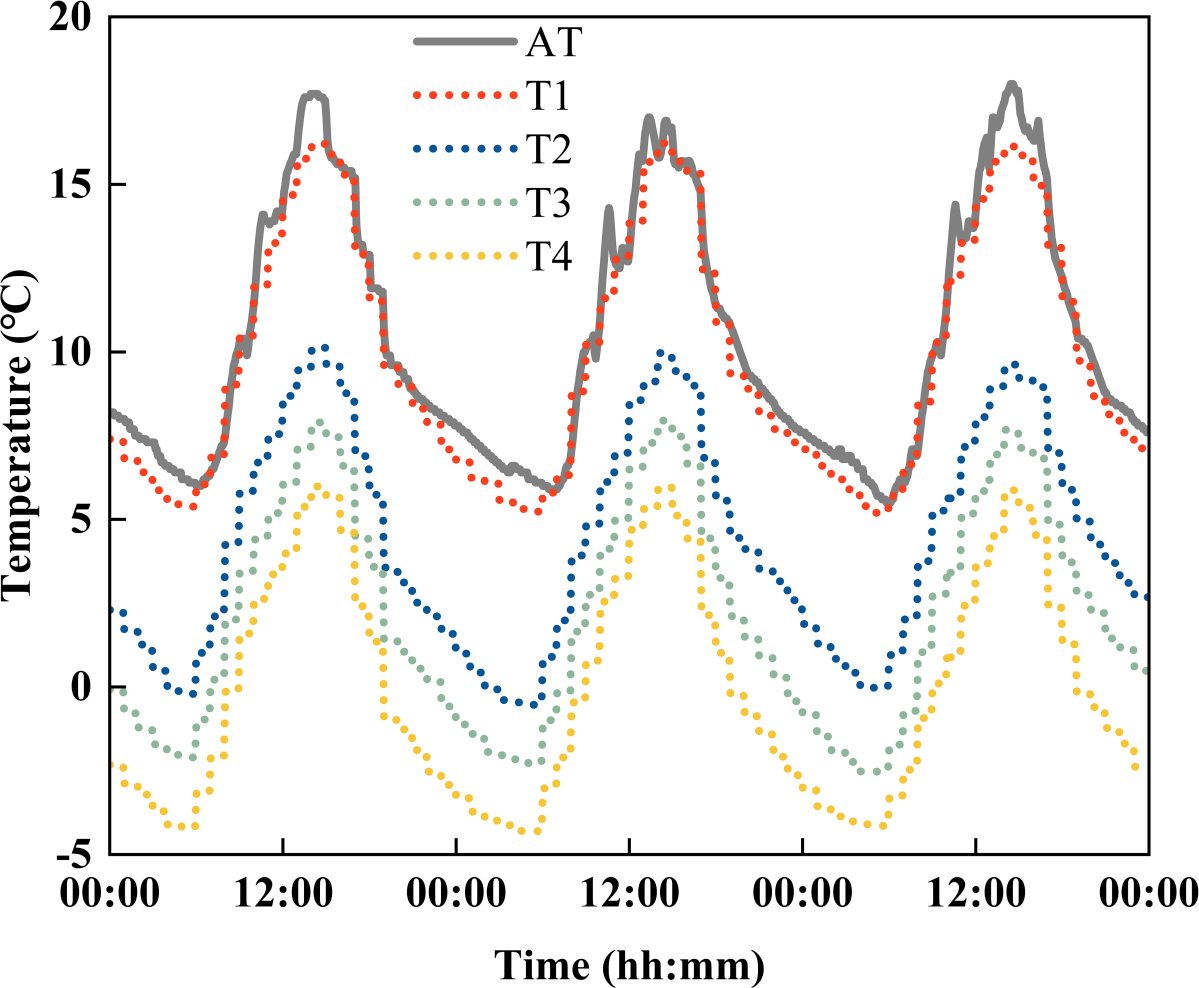
Measured daily temperature dynamics in controlled-environment phytotrons during low-temperature stress treatments (data were collected from March 6 to 8, 2019). AT indicates ambient temperature.

The daily temperature dynamics in the controlled-environment phytotrons during the treatment period are shown in **Figure 1**, which conforms to the daily variation pattern in the natural state. In addition, to keep the factors other than the temperature in the four controlled-environment phytotrons as consistent as possible, inlet and outlet fans were installed at the front and rear of the phytotrons for relatively moderate gas exchange with the surrounding environment, and halogen lamps were used for supplemental lighting to ensure that the growth of wheat was not limited by light. The light intensity in the phytotrons was 1380 μmol m^−1^ s^−1^ and 240 μmol m^−1^ s^−1^ at noon on sunny and cloudy days, respectively; thus, the experimental conditions enable the negative effects of light and CO_2_ to be avoided.

### 2.2 Sampling and measurements

For all treatments, the kernels of plants in 9 pots (each replicate constituted 3 pots) were harvested at maturity, and after being air dried, the kernels were ground into whole wheat flour for the test of mineral components.

Samples were subjected to high-temperature digestion with an HNO_3_/HClO_4_ acid mixture (Johnson and Ulrich, 1959), and the mineral component (P, K, Ca, Fe, and Zn) contents were determined by inductively coupled plasma emission spectrometry (ICP−OES 710; Agilent Technologies, USA) (Tracy et al., 1991).

The accumulation of mineral components in wheat grains was calculated by multiplying the mineral component concentration by the grain weight. The wheat grain weight under different low-temperature treatments is shown in **Supplementary Figure S1**.

### 2.3 Comprehensive evaluation of mineral nutrition quality

The concept of fuzzy set theory was employed to evaluate the comprehensive nutrition quality of wheat grains after low-temperature treatment. The fuzzy integrated evaluation method is a comprehensive evaluation method that converts a qualitative evaluation into a quantitative evaluation based on the membership grade theory of fuzzy mathematics; i.e., fuzzy mathematics is used to make an overall evaluation of food or objects that are subject to multiple factors. The fuzzy membership function value is a comprehensive index that can not only indicate relative advantages and disadvantages but also reflect the comprehensive characteristics of the overall levels of multiple constituents, making it an effective method for evaluating nutritional quality. The value of each membership function is calculated and averaged, and the larger the average value is, the better the comprehensive nutritional quality (Zhao et al., 2022). The membership function values are calculated as follows:


(1) $$U\left(X_{ij}\right)=\frac{X_{ij}-min\left(X_{j}\right)}{max\left(X_{j}\right)-min\left(X_{j}\right)}$$

(2) $$U\left(X_{ij}\right)=1-\frac{X_{ij}-min\left(X_{j}\right)}{max\left(X_{j}\right)-min\left(X_{j}\right)}$$

(3) $$X_{i}=\frac{\sum^{}U\left(X_{ij}\right)}{n}$$

where *X_ij_* is the measured value of indicator j of treatment i, and *max(X_j_)* and *min(X_j_)* are the maximum and minimum values of the indicator, respectively. *U(X_ij_)* is the membership function value of indicator j of treatment i, *X_i_* is the average membership function value of treatment i, and n is the number of samples measured.

When the indicators are positively correlated with nutritional quality, Eq. (1) can be used to calculate their membership function values, and when the indicators are negatively correlated with nutritional quality, Eq. (2) can be used to calculate their membership function values.

### 2.4 Statistical analysis

Statistical analysis (ANOVA) of data from the two-year trial was performed using SPSS 24.0 software (SPSS Inc., Chicago, IL, USA), and multiple comparisons between treatments (*p*< 0.05) were performed using Duncan’s method. OriginPro 2021 software (OriginLab, Wellesley Hills, MA, USA) was used to graph the mineral component content and accumulation.

## 3 Results

### 3.1 Effects of low-temperature stress on major mineral components in wheat grains

#### 3.1.1 Effects of low temperature on the content of major mineral components

**Figure 2:**
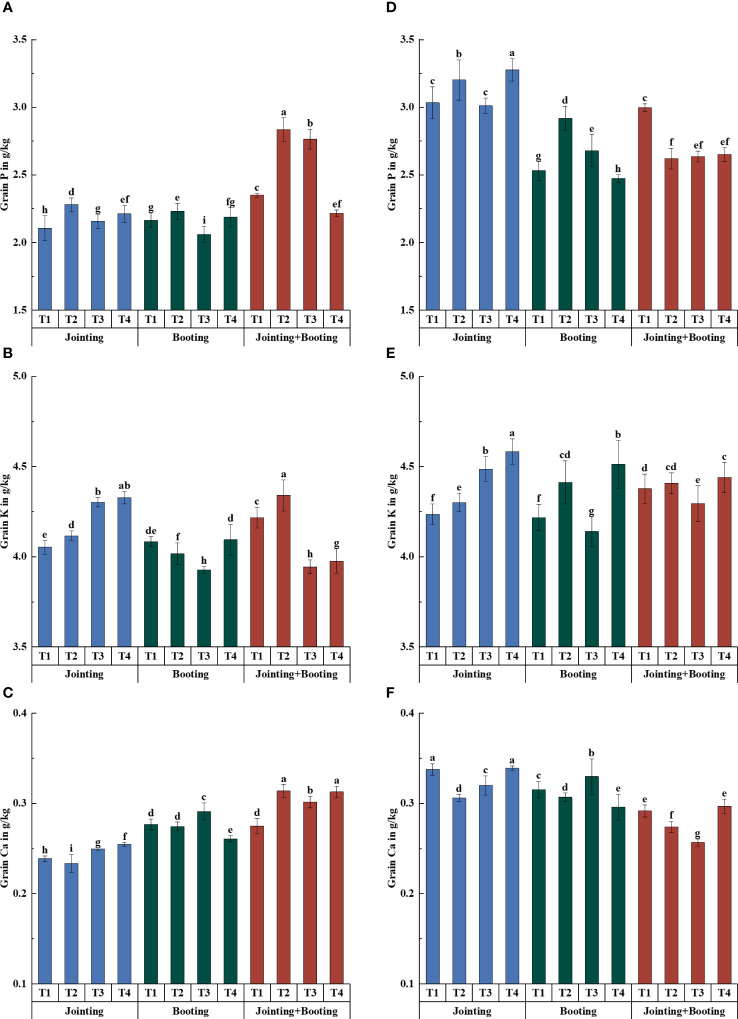
Contents of phosphorus (P), potassium (K), and calcium (Ca) in grains under different low-temperature treatments in Yangmai16 **(A−C)** and Xumai30 **(D−F)**. Vertical bars represent the standard deviation of the mean. Different lowercase letters indicate significant differences at *p*<0.05.

**Figure 3:**
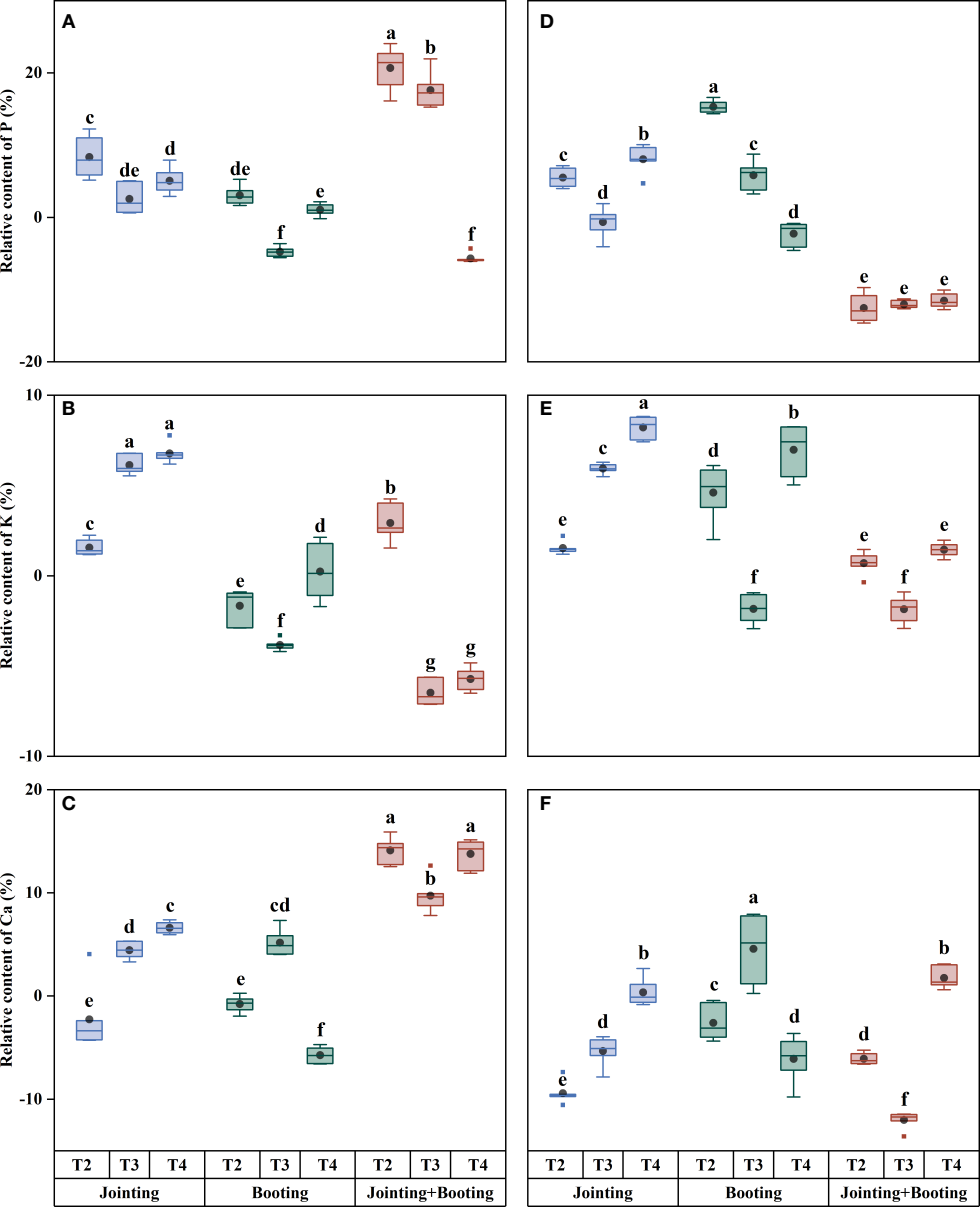
Relative contents of phosphorus (P), potassium (K), and calcium (Ca) in grains under different low-temperature treatments in Yangmai16 **(A−C)** and Xumai30 **(D−F)**. The horizontal lines show the maximum and minimum values; the middle line shows the median; the upper and lower edges of the boxes show the 75th and 25th percentiles, respectively; the black circles represent the mean values, and the discrete squares represent the outliers. Different lowercase letters indicate significant differences at *p*<0.05.

Different treatment periods and low-temperature levels had significant effects on the content of major mineral components in wheat grains. The grain P content ranged from 2.06−3.28 g/kg, and the relative content ranged from −12.54% to 20.71% (**Figures 2**, **3**). Under low-temperature treatment during the jointing stage, the P content of both Yangmai16 and Xumai30 decreased first and then increased with decreasing temperature (from T2 to T4). The variation in the grain P content of Yangmai16 in the booting stage was consistent with the P variation under low-temperature treatment applied at the jointing stage, while the trend of Xumai30 was different from that of Yangmai16, showing a single-peak curve distribution with the highest content at T2 (0/5/10°C, T_min_/T_avg_/T_max_). The grain P content relative to temperature showed opposite trends between cultivars under low-temperature treatment during the combined stages (jointing and booting) (**Figures 2A, D**). In addition, the weak (T2) and medium (T3, −2/3/8°C, T_min_/T_avg_/T_max_) low-temperature levels during the jointing stage produced certain acclimation effects in the cold-sensitive cultivar (Yangmai16), which could mitigate the effects of low-temperature stress during the booting stage on grain P content to some extent. In general, the magnitude of changes in the grain P content of Yangmai16 and Xumai30 (except for at T2) under low-temperature treatment during the combined stages (jointing and booting) was greater than that during either single stage (**Figure 3**).

Both wheat cultivars showed a trend of increasing grain K content with decreasing temperature under low-temperature treatment applied at the jointing stage, while the variation in the grain K content of both cultivars showed a trend of first decreasing and then increasing with decreasing temperature under low-temperature treatment during the booting stage and combined stages. The lowest content was recorded at T3. Additionally, T2 produced certain acclimation effects in Yangmai16 under low-temperature treatment during the jointing stage (**Figures 2B, E**).

For grain Ca content, the trend of both wheat cultivars under low-temperature treatment during the jointing stage and combined stages was consistent with that of K content, and under low-temperature treatment applied at the booting stage, Ca content first increased and then decreased with decreasing temperature (from T2 to T4). In addition, for Yangmai16, T2, T3, and T4 (−4/1/6°C, T_min_/T_avg_/T_max_) all had acclimation effects on grain Ca content. However, Xumai30 only showed an acclimation effect under the strong low-temperature level (T4).

#### 3.1.2 Effects of low temperature on the accumulation of major mineral components

**Figure 4:**
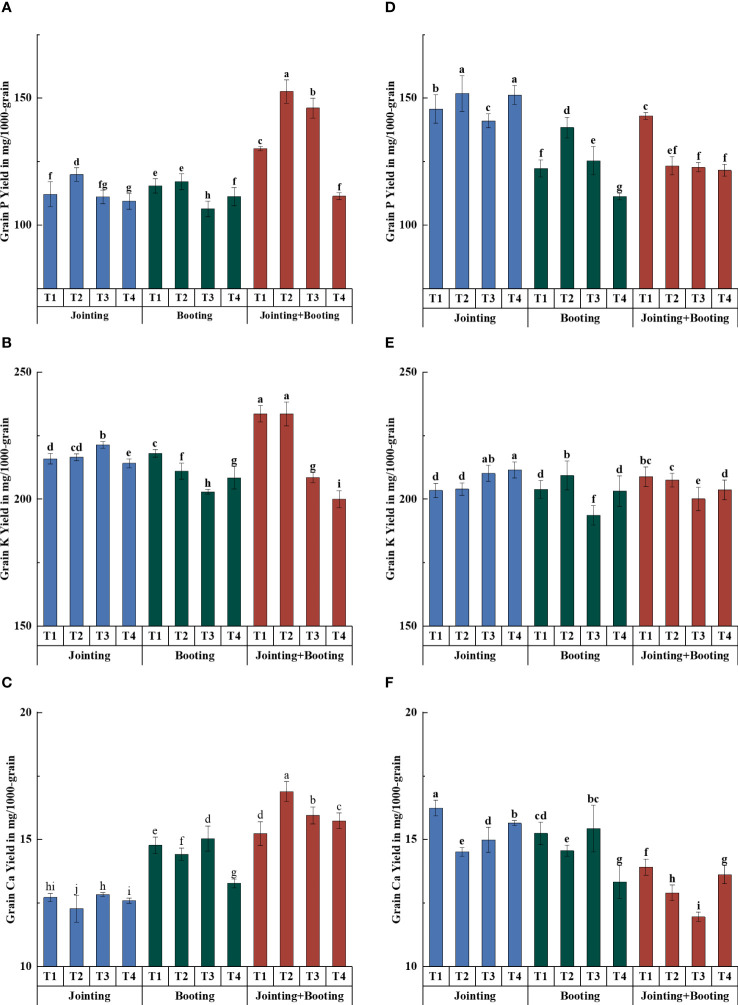
Accumulation of phosphorus (P), potassium (K), and calcium (Ca) in grains under different low-temperature treatments in Yangmai16 **(A−C)** and Xumai30 **(D−F)**. Vertical bars represent the standard deviation of the mean. Different lowercase letters indicate significant differences at *p*<0.05.

**Figure 5:**
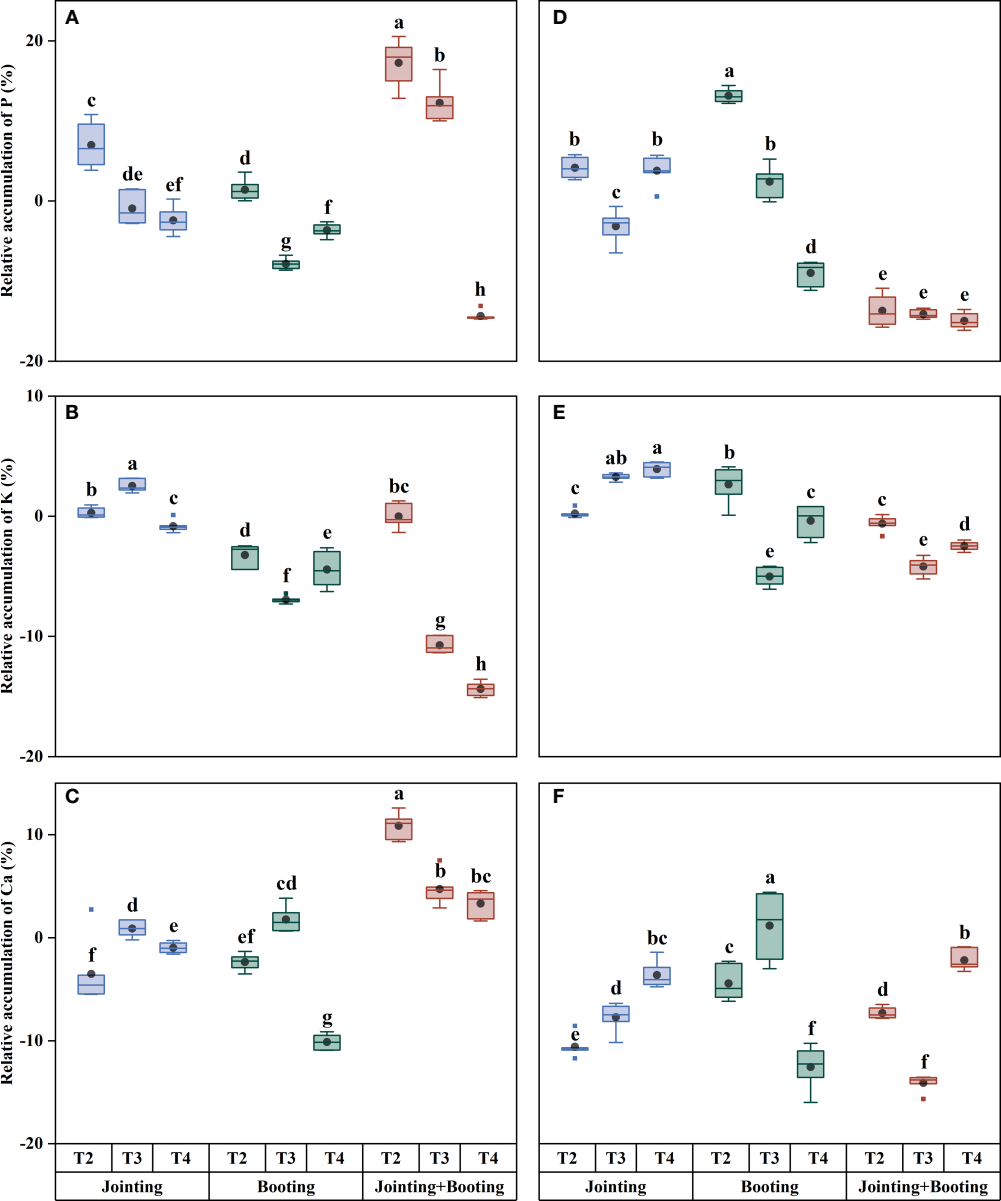
Relative accumulation of phosphorus (P), potassium (K), and calcium (Ca) in grains under different low-temperature treatments in Yangmai16 **(A−C)** and Xumai30 **(D−F)**. The horizontal lines show the maximum and minimum values; the middle line shows the median; the upper and lower edges of the boxes show the 75th and 25th percentiles, respectively; the black circles represent the mean values, and the discrete squares represent the outliers. Different lowercase letters indicate significant differences at *p*<0.05.

The effects of different treatment periods and low-temperature levels on the accumulation and content of major mineral components in wheat grains were consistent (**Figures 2**–**5**). The different changes were mainly concentrated in Yangmai16. Under low-temperature treatment applied at the jointing stage, the accumulation of P in Yangmai16 showed a downward trend with decreasing temperature, and the accumulation of K and Ca showed a rising and then falling trend with decreasing temperature (from T2 to T4). The accumulation of P in Xumai30 and K and Ca in Yangmai16 showed a downward trend with decreasing temperature under the combined stage (jointing and booting) treatment. Yangmai16, a cold-sensitive cultivar, has a less predictable response to low-temperature stress.

### 3.2 Effects of low-temperature stress on trace mineral components in wheat grains

**Figure 6:**
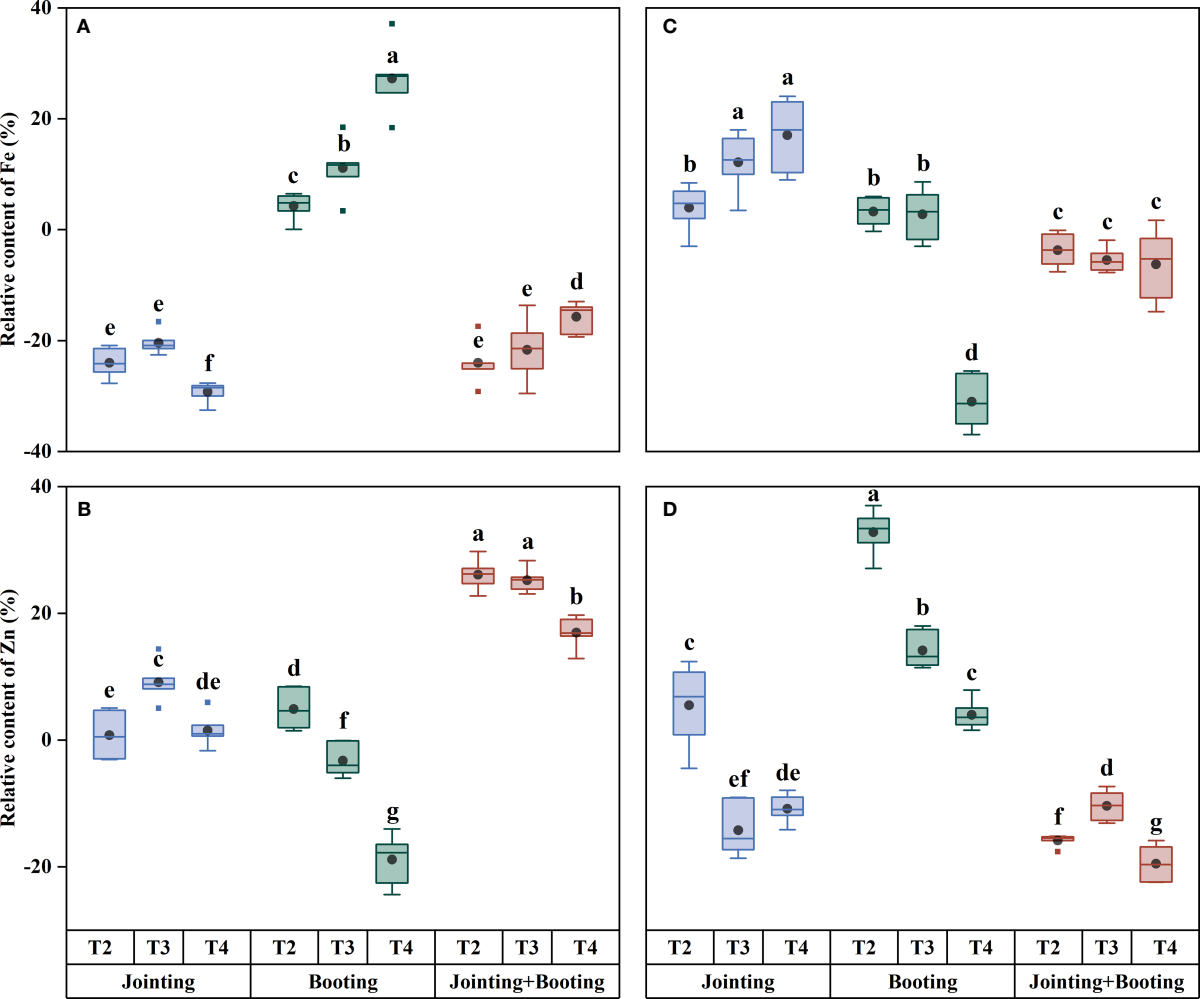
Relative contents of iron (Fe) and zinc (Zn) in grains under different low-temperature treatments in Yangmai16 **(A, B)** and Xumai30 **(C, D)**. The horizontal lines show the maximum and minimum values; the middle line shows the median; the upper and lower edges of the boxes show the 75th and 25th percentiles, respectively; the black circles represent the mean values, and the discrete squares represent the outliers. Different lowercase letters indicate significant differences at *p*<0.05.

**Figure 7:**
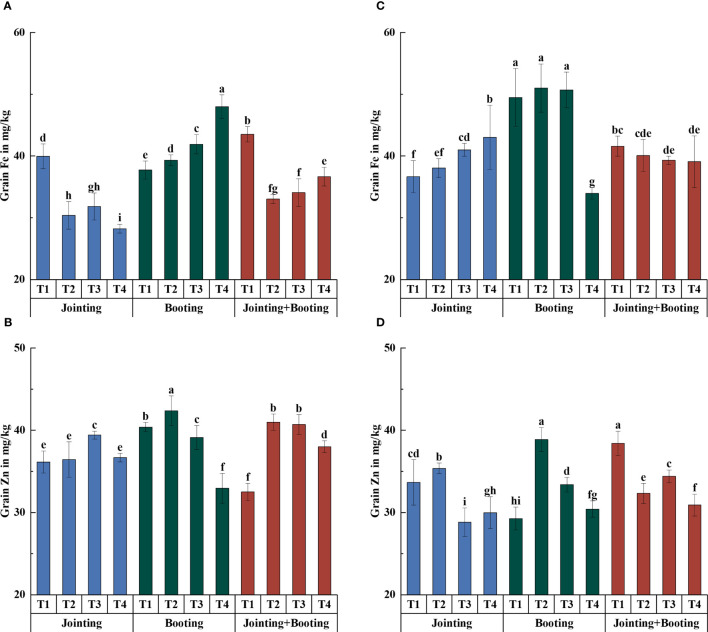
Contents of iron (Fe) and zinc (Zn) in grains under different low-temperature treatments in Yangmai16 **(A, B)** and Xumai30 **(C, D)**. Vertical bars represent the standard deviation of the mean. Different lowercase letters indicate significant differences at *p*<0.05.

**Figure 8:**
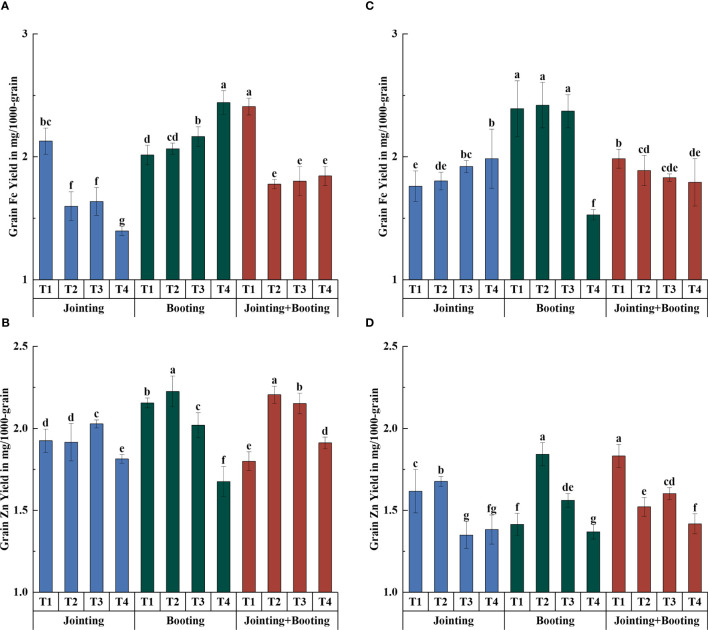
Accumulation of iron (Fe) and zinc (Zn) in grains under different low-temperature treatments in Yangmai16 **(A, B)** and Xumai30 **(C, D)**. Vertical bars represent the standard deviation of the mean. Different lowercase letters indicate significant differences at *p*<0.05.

**Figure 9:**
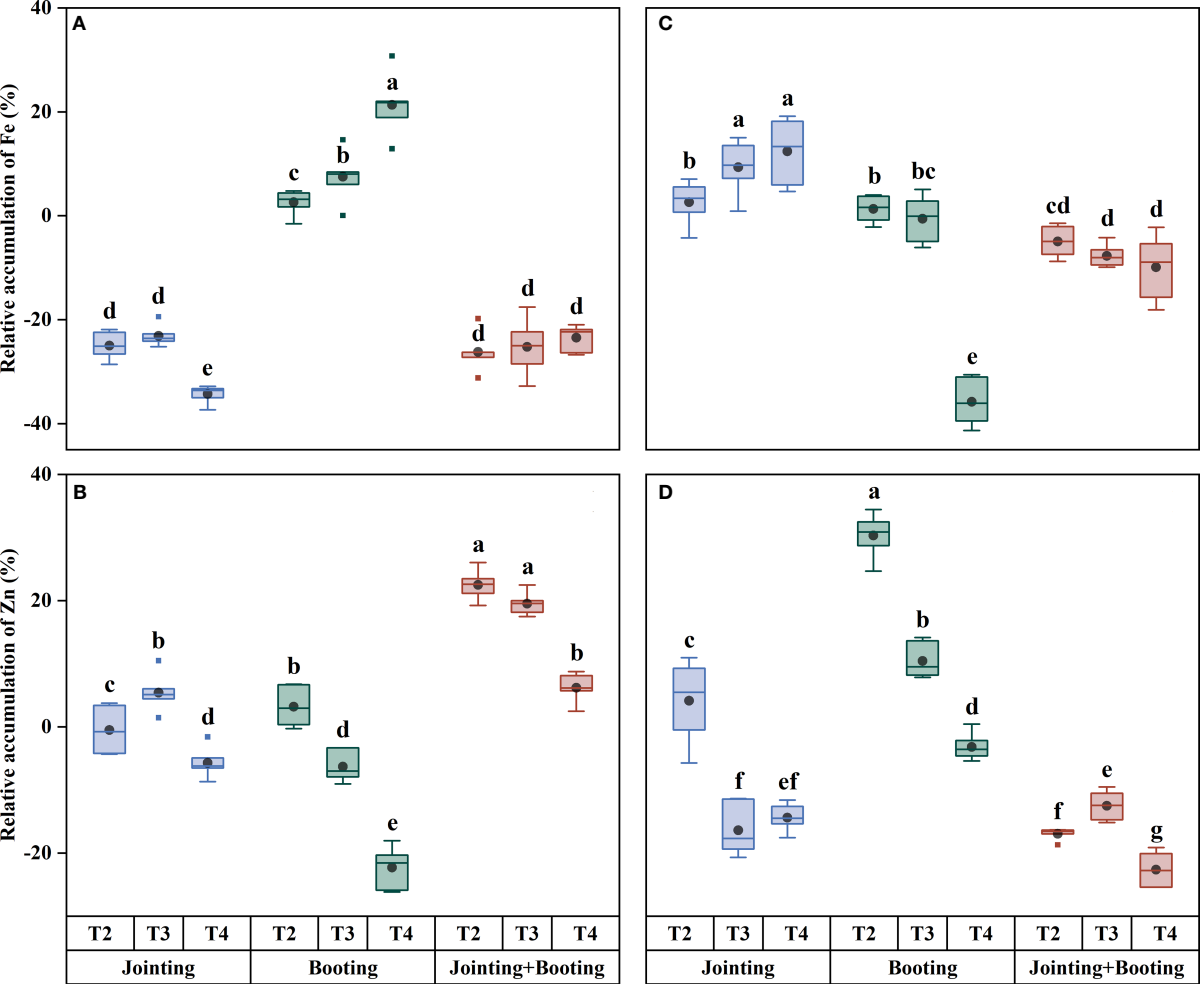
Relative accumulation of iron (Fe) and zinc (Zn) in grains under different low-temperature treatments in Yangmai16 **(A, B)** and Xumai30 **(C, D)**. The horizontal lines show the maximum and minimum values; the middle line shows the median; the upper and lower edges of the boxes show the 75th and 25th percentiles, respectively; the black circles represent the mean values, and the discrete squares represent the outliers. Different lowercase letters indicate significant differences at *p*<0.05.

With values ranging from −31.40% to 32.74% (**Figures 3**, **6**), the content of trace mineral components (Fe, Zn) in the wheat grains was typically more affected than the content of major mineral components by low temperature. Additionally, the trend of trace mineral accumulation under low temperature was essentially consistent with that of the overall mineral content (**Figures 6**–**9**). For grain Fe content, the variation ranged from 28.24−51.02 mg/kg (**Figures 7A, C**). Under low-temperature treatment during the jointing stage, the grain Fe content in Yangmai16 increased first and then decreased with decreasing temperature (from T2 to T4), while in Xumai30, it increased. With decreasing temperature, the Fe content showed opposite trends between the cultivars under low-temperature treatment applied at the booting stage and combined stage (jointing and booting). Moreover, Yangmai16 was more impacted by low-temperature stress than Xumai30 (**Figure 6**), and the strong low-temperature level (T4) had an acclimation effect on the Fe content of Xumai30.

For grain Zn content, the values under low temperature ranged from −19.51% to 32.74%. The trend of Zn content in Yangmai16 under the jointing stage treatment was consistent with that of Fe, while the Zn content in Xumai30 showed an opposite trend from that in Yangmai16. Under the booting stage treatment, the grain Zn contents for Yangmai16 and Xumai30 showed the same trend and were the highest at the weak low-temperature level (T2). Under the combined stage treatment, the trend of Zn content in Yangmai16 was consistent with that at the booting stage, while in Xumai30, the Zn content increased first and then decreased with decreasing temperature (from T2 to T4). In addition, different low-temperature levels (T2, T3, and T4) all had acclimation effects on the Zn content of Yangmai16.

In summary, there were differences in the effects of low-temperature stress on the content and accumulation of mineral components in wheat grains during different periods. Under T2, low-temperature treatment applied at the combined stage (jointing and booting stages) had the greatest impact on the content and accumulation of all mineral components (except K accumulation) in Yangmai16 and the accumulation of P and Fe in Xumai30; low-temperature treatment applied at the jointing stage had the greatest impact on the content and accumulation of Fe and Ca in Xumai30, while the other treatments were most affected by low temperature during the booting stage. Under T3, the jointing stage treatment had the greatest effect on the K content and the Fe and Zn content and accumulation in Xumai30; only the K accumulation in Xumai30 was most affected by low temperature during the booting stage, while the remaining treatments were all most affected by low temperature applied at the combined stage. Under T4, the K content, Fe content and accumulation of Yangmai16 and the K content and accumulation of Xumai30 were most affected by low temperature during the jointing stage, and the Zn content and Ca and Zn accumulation in Yangmai16 and the Ca and Fe content and accumulation in Xumai30 were most affected by low temperature during the booting stage. In addition, according to the ANOVA results (**Table 2**), the effects of different treatment stages (S), different low-temperature intensities (T), and their interactions (T×S) on the content and accumulation of mineral components in grains were highly significant (*p*< 0.01).

**Table 2:** Effects of different low-temperature intensities (T), stages (S), and their interactions (T×S) on mineral components of wheat.

Cultivar	Source of Variation	Content					Accumulation				
		P	K	Ca	Fe	Zn	P	K	Ca	Fe	Zn
Yangmai16	S	**	**	**	**	**	**	**	**	**	**
	T	**	**	**	**	**	**	**	**	**	**
	S×T	**	**	**	**	**	**	**	**	**	**
Xumai30	S	**	**	**	**	**	**	**	**	**	**
	T	**	**	**	**	**	**	**	**	**	**
	S×T	**	**	**	**	**	**	**	**	**	**

** indicates significance at p < 0.01.

### 3.3 Comprehensive evaluation of the effects of low temperature on mineral components in wheat grains

The membership function model was used to calculate the eigenvalues of the mineral component content in wheat grains under different low-temperature treatments, and the average value of the eigenvalues was sorted as a comprehensive score to comprehensively evaluate the mineral nutrition in grains (**Table 3**). Under low-temperature treatment during the jointing stage, the comprehensive score of the mineral component content in Yangmai16 at T1 was 0.278, which was lower than those at T3 (0.422) and T4 (0.370) but higher than that at T2 (0.249). However, the results of the comprehensive evaluation of the minerals in Xumai30 were different from those for Yangmai16, and the comprehensive scores for Xumai30 at T2, T3, and T4 were greater than those at T1, indicating that the nutritional value of Xumai30 was enhanced after low-temperature stress. Under the booting stage treatment, the comprehensive scores of grain mineral components in Yangmai16 compared to T1 increased at the weak low-temperature level (T2) and decreased significantly at the medium (T3) and strong (T4) low-temperature levels. With decreasing temperature (from T2 to T4), the comprehensive scores of minerals in Xumai30 decreased, although the comprehensive scores of minerals at T2 and T3 were still greater than those at T1. Under the combined stage treatment (jointing and booting stages), the comprehensive mineral scores for Yangmai16 at T2 and T3 were 0.820 and 0.584, respectively, ranking first and second among the mineral scores, indicating that the mineral component content in the wheat grains was relatively high and that the mineral nutrition was better, while the comprehensive scores of mineral components in Xumai30 all decreased significantly.

**Table 3:** Effects of different low-temperature intensities and stages on the membership function values of mineral components in Yangmai16 and Xumai30 and comprehensive evaluation.

Cultivar	Treatment		P	K	Ca	Fe	Zn	Comprehensive evaluation value	Order
Yangmai16	Jointing	T1	0.059	0.303	0.067	0.593	0.368	0.278	11
		T2	0.283	0.457	0.000	0.108	0.398	0.249	12
		T3	0.126	0.905	0.198	0.181	0.700	0.422	7
		T4	0.195	0.969	0.263	0.000	0.422	0.370	10
	Booting	T1	0.133	0.379	0.539	0.481	0.798	0.466	5
		T2	0.218	0.215	0.512	0.562	1.000	0.501	3
		T3	0.000	0.000	0.718	0.692	0.670	0.416	8
		T4	0.163	0.403	0.341	1.000	0.043	0.390	9
	Jointing + booting	T1	0.372	0.701	0.518	0.773	0.000	0.473	4
		T2	1.000	1.000	1.000	0.242	0.859	0.820	1
		T3	0.907	0.039	0.849	0.295	0.831	0.584	2
		T4	0.200	0.117	0.988	0.427	0.558	0.458	6
Xumai30	Jointing	T1	0.697	0.214	0.986	0.159	0.482	0.508	7
		T2	0.907	0.361	0.599	0.239	0.652	0.552	4
		T3	0.671	0.782	0.768	0.413	0.000	0.527	5
		T4	1.000	1.000	1.000	0.532	0.116	0.729	2
	Booting	T1	0.072	0.174	0.711	0.910	0.045	0.383	8
		T2	0.555	0.614	0.609	1.000	1.000	0.756	1
		T3	0.258	0.000	0.888	0.981	0.455	0.516	6
		T4	0.000	0.840	0.479	0.000	0.160	0.296	11
	Jointing + booting	T1	0.651	0.533	0.426	0.448	0.954	0.603	3
		T2	0.183	0.602	0.210	0.360	0.349	0.341	10
		T3	0.202	0.351	0.000	0.313	0.556	0.284	12
		T4	0.221	0.676	0.488	0.300	0.208	0.379	9

## 4 Discussion

Wheat is one of the world’s most important staple food crops, feeding billions of people worldwide and supplying more than 45% of global energy (Zhong et al., 2018; Zhu et al., 2018). Although wheat is a winter crop, it is still sensitive to low temperatures, especially from the jointing stage to the booting stage (Barton et al., 2014). Due to climate change, short episodes of low-temperature stress events occur frequently in late spring and pose a serious threat to wheat yield (Zheng et al., 2015; Ji et al., 2017; Xiao et al., 2018) and quality (Liu et al., 2019; Zhang et al., 2021). However, most previous studies have been primarily concerned with the effects of heat stress on crop production, and low-temperature injury to crops, especially on the mineral nutrition of wheat grains, has usually been ignored.

### 4.1 Responses of mineral components in wheat grains to low-temperature stress

Minerals are involved not only in plant energy metabolism, enzymatic reactions, and osmoregulation, which are the material basis of plant life activities but also in various biochemical reactions in human cells that are essential for the prevention of diseases and maintenance of health in humans (Gharibzahedi and Jafari, 2017). The accumulation of mineral components in wheat grains is a complex physiological process involving the collaboration of multiple organs and tissues performing different functions, such as root uptake, above ground transport, vegetative organ re-mobilization, and grain storage (Gupta et al., 2021). The content and accumulation of mineral components in grains at final maturity are determined by a combination of genotype, ecological environment, and cultivation practices (Caldelas et al., 2023).

However, the current status of climate change is more drastic than ever before, and environmental factors are exerting more prominent influences on the mineral nutritional quality of crops, with considerable direct and indirect effects (Soares et al., 2019). Compared with warming or high temperatures, the effects of low-temperature stress on wheat are often neglected (Yue et al., 2016; Xiao et al., 2018). Tachibana found that when the root system of cucumber was exposed to low temperature, the P content in the leaves decreased significantly, and the K and Ca contents decreased slightly. The stems showed a similar pattern as the leaves, but low temperature affected the mineral components in the stems to a lesser extent than those in the leaves (Tachibana, 1982). Rivero et al. analyzed the uptake and distribution of Fe in tomato and watermelon at different cultivation temperatures (10°C, 25°C, and 35°C) and found that low temperature reduced the uptake of Fe by watermelon, which may be related to a decrease in the activity of enzymes involved in Fe metabolism (Rivero et al., 2003). Kalisz et al. applied a 7-day low-temperature treatment at 4°C to different varieties of cauliflower during the seedling stage, and the response of some varieties to low temperature showed that the contents of N, P, Ca, S, Mg, Na, B, Cu, Zn, and Pb increased, but the contents of Mo and Cr decreased (Kalisz et al., 2018). Furthermore, warming at 3°C increased the contents of Cu, Zn, and Fe in potato leaves by 25%, 27%, and 24%, respectively, while decreasing the contents of Cd, Pb, Fe, Zn, and Cu in tubers by 27%, 55%, 41%, 29%, and 23%, respectively (Li et al., 2012). According to field research dynamics, most recent studies on the effects of low temperature on plant mineral components are focused on horticultural crops, and relatively few studies have been conducted on field crops. The few studies on the effects of low temperature on the mineral component content of field crops are focused on the seedling stage (Edwards and Kamprath, 1974) and mainly consider the organs of the plant, such as roots, stems, and leaves; in contrast, there are no such studies on cereal grains, which are more closely connected to human nutrition and food security. Therefore, it is important to carry out studies on the effects of low temperature on mineral components in wheat grains.

In the present study, we found that, compared with trends in the control, the Fe content of Yangmai16 under low-temperature treatments during the jointing stage and combined stages and the P, Fe, and Zn contents of Xumai30 under low-temperature treatments during the combined stages all decreased, which was consistent with the results of Miyasaka and Gonzalez-Fuentes in winter wheat and strawberry experiments under different RZTs (Miyasaka and Grunes, 1997; Gonzalez-Fuentes et al., 2016). Minerals in crop plants mainly come from root uptake, and at present, the reasons for the decrease in mineral uptake by plant roots due to low-temperature stress are mainly explained from two angles. On the one hand, low temperature directly inhibits root growth and development and cell differentiation and elongation so that root growth slows or even stops, which increases the transport resistance of water and nutrients into the root system and limits the absorption efficiency and hydraulic conductivity of the root system. In addition, low temperature in the rhizosphere can be sensed by the root system and transmitted upward via plant signals (hormones, ions, etc.), indirectly affecting above-ground physiological and biochemical processes (Hong et al., 2017). On the other hand, according to Li et al., temperature also affects soil physicochemical properties, leading to changes in the solubility of minerals in the soil and an imbalance of mineral component inputs and outputs in the soil−plant system, thus changing the distribution characteristics of mineral components in crop organs and significantly affecting the enrichment levels of minerals in the edible fraction of crops (Li et al., 2012). Moreover, roots, serving as vital organs for nutrient absorption and transport, are typically more susceptible to low temperature than other vegetative organs, such as leaves and stems (Kul et al., 2020). Based on the findings of this study, we propose that the impact of pre-anthesis low temperature on root-mediated mineral nutrient uptake will eventually manifest in the enrichment levels of mineral nutrients in mature grains. Consequently, enhancing root resilience to low temperature is crucial for preserving the mineral nutrition quality of wheat grains.

The effects of low-temperature stress on the nutritional quality of wheat depend not only on the temperature level and duration of stress but also on the stage of wheat growth and development at which the stress occurs. Previous studies have shown that compared with the low-temperature duration, the low-temperature level had a more significant effect on the nutritional quality, quality of appearance, and processing quality of wheat, and these quality parameters were identified to be more sensitive to low temperature during the booting than the jointing stage (Liu et al., 2019). Hood et al. concluded that the uptake of mineral components (P, K, Ca, Fe, and Zn) by snapdragon showed a significant quadratic function response with increasing RZTs (8−36°C) and that the effects of low temperature occurring at different developmental stages on the uptake and accumulation of mineral components in plants were different (Hood and Mills, 1994). In this study, we also found that the effects of low-temperature stress on mineral components in wheat grains were related to the low-temperature treatment period. The P, K, and Zn contents of Yangmai16 and the K and Fe contents of Xumai30 under the jointing stage treatment, the Fe content of Yangmai16 and the Zn content of Xumai30 under the booting stage treatment, and the Ca and Zn contents of Yangmai16 under the combined-stage low-temperature treatment all increased compared to those of the control, which is similar to some results of Kalisz and Tachibana (Tachibana, 1982; Kalisz et al., 2018). The effects of low-temperature stress on mineral component accumulation in this study were similar to its effects on content, with the difference that mineral component accumulation decreased to a greater degree under low temperature. Typically, low temperature reduced the 1000-grain weight more significantly (Ji et al., 2017), and grain mineral component accumulation is determined by both the mineral component content and grain weight, which indicates that the content of mineral components plays a major role in determining mineral component accumulation under most low-temperature treatments. Furthermore, this research found that under different low-temperature treatment periods, the content and accumulation of wheat grain mineral components responded differently to different low-temperature intensities. Under T2, the content and accumulation of mineral components were more affected by low-temperature treatment at the combined stages, followed by the booting stage; under T3, the mineral content and accumulation were more affected by low-temperature treatment at the combined stages, followed by the jointing stage; under T4, the content of mineral components was most affected by low-temperature treatment at the combined stages, and accumulation was more affected by low-temperature treatment at the combined stages and booting stage. In general, most mineral nutrients of the cold-sensitive cultivar were more sensitive to low temperature during the combined stages, while the mineral nutrients of the cold-tolerant cultivar were more sensitive to low temperature during the booting stage. Thus, selecting suitable cold-tolerant cultivars based on the specific climatic conditions in each region is an effective method to improve the nutritional quality of wheat under low-temperature stress conditions in the future.

Interestingly, the role of various minerals in plants varies, so the reasons for the increases in the content of different minerals in the present study may not be consistent, but they may all be related to the stress resistance of plants. Ramalho et al. found, by analyzing the dynamics of mineral component contents in the leaves of different cold-tolerant coffee varieties under low temperature and the key enzyme activities of related physiological processes, that N, Fe, Zn, Mn, and Cu may contribute positively to the adaptation of coffee to low temperature and could be a strong basis for the assessment of cold tolerance in varieties (Ramalho et al., 2013). P is a component of key organic compounds such as nucleic acids and phospholipids and influences photosynthesis, respiration, and various cellular metabolic processes in plants, and K is involved in regulating biochemical processes related to protein synthesis, carbohydrate metabolism, and enzyme activation, in addition to maintaining physiological processes such as osmotic balance, stomatal regulation, and photosynthesis. The increases in P and K content may be due to the ability of P and K to reduce the water potential in cells, maintain normal cell metabolism, and enhance the stability and frost resistance of wheat seedling membranes in low-temperature environments (Ankit et al., 2022; Xu et al., 2022). Ca not only maintains the stability of the cell wall and cell membrane structure and function but also acts as a secondary messenger to transmit information, which is closely associated with plant immunity (Thor, 2019). Elevated Ca contents may be a result of a rapid increase in Ca^2+^ concentration under low temperature, which may induce the trans membrane transmission of Ca^2+^ signals in response to the adverse effects of low-temperature (Kleiner et al., 2022). Zn is an activator and essential element for many enzymes, and protein synthesis is hindered when plants are deficient in Zn, leading to the accumulation of amino acids (Shrivastav et al., 2020). The elevated Zn content in wheat grains caused by low-temperature may be due to the presence of Zn in super-oxide dismutase (SOD) and catalase (CAT), and an increase in Zn content can enhance the antioxidant capacity of plant cells (Batova et al., 2021). Increases in Fe content may be due to cold-induced expression of Fe uptake-related genes, causing the root tips to actively secrete reducing substances such as H^+^, organic acids, and phenols into the root environment, thereby increasing the solubility of external Fe (Zhang et al., 2019).

### 4.2 Comprehensive evaluation of the effects of low-temperature stress on the mineral nutritional value of wheat grains

Although people’s health awareness regarding nutritional balance has gradually improved with the advancement of society and living conditions, the intake of micronutrients is still generally insufficient. Importantly, the severity of this ‘hidden hunger’ is being worsened by climate change, and it primarily affects regions where cereals such as wheat and rice are the staple foods (Park et al., 2019; Soares et al., 2019; Mirzabaev et al., 2023). As a result, it is crucial to evaluate the nutritional value of the minerals in wheat. However, most previous studies have focused on the trends in mineral component content without a systematic scientific assessment of the nutritional value of the mineral components. In consideration of the one-sidedness and limitations of single-indicator analysis, this study introduces the membership function evaluation method for the comprehensive evaluation of the nutritional quality of cereal grains. This method can comprehensively evaluate foods or objects subject to multiple factors, and the membership function value can not only express comparative superiority or inferiority but also reflect the overall level of components based on comprehensive characteristics, which is an effective method for evaluating nutritional quality with a certain level of objectivity and accuracy (Zhao et al., 2022).

The membership function method is commonly used for multi-index evaluation and has been widely applied in research on stress resistance in field crops and horticultural crops (Liu et al., 2015; Song et al., 2017; Weng et al., 2021; Chen et al., 2023). Liu et al. used the membership function method to evaluate drought resistance for 10 agronomic traits in wheat (Liu et al., 2015). Based on multiple agronomic traits and antioxidant enzyme activities, Song et al. used the membership function value of drought resistance (MFVD) and drought resistance index (DI) to comprehensively evaluate the drought resistance of 47 wheat germplasm samples under water deficit and well-irrigated conditions, which facilitated the comprehensive evaluation of crop stress resistance indicators and screening of stress-resistant varieties (Song et al., 2017). This study found that the comprehensive evaluation values for mineral components in Yangmai16 (under T3 and T4 during the jointing stage, T2 during the booting stage, and T2 and T3 during combined stages) and Xumai30 (under all treatments during the jointing stage and under T2 and T3 during the booting stage) were higher than T1, indicating some improvement in the mineral nutritional value of wheat grains, while the remaining treatments exhibited decreased comprehensive evaluation values compared to the control, which indicated that low-temperature stress had extremely adverse effects on mineral components. In addition, the comprehensive evaluation value of mineral components in Yangmai16 under low-temperature treatment during the combined stages was greater than those under treatment during the jointing stage and the booting stage, while the comprehensive evaluation value of Xumai30 under low-temperature treatment during the combined stages was relatively low. This indicates that low temperature applied at the jointing stage produced certain acclimation effects in Yangmai16, which alleviated the harm caused by low temperature during the booting stage. However, after experiencing low temperatures during the combined stages, the impacts of low temperatures on the mineral component contents of Xumai30 were exacerbated. Future production strategies should include paying particular attention to the effects of low-temperature stress on wheat during these periods, and precautions should be taken in advance.

## 5 Conclusion

To date, more studies have considered the impact of low temperature on grain protein and starch, but the effects of low temperature on mineral components should not be ignored. In this study, different low-temperature regimes were designed to investigate the effects of low temperature on the mineral nutrients in different wheat cultivars. Our results suggested that low temperature increased the contents of P, K, Ca, and Zn in Yangmail6 and K in Xumai30, while decreased the mineral accumulation, except for P, Ca, and Zn in Yangmai16. Minerals (P, K, Ca, and Zn) in the cold-sensitive cultivar were more susceptible to low temperature during the combined stage, while minerals (K, Fe, and Zn) in the cold-tolerant cultivar were more susceptible to low temperature during the booting stage. In addition, trace minerals (Fe and Zn) in wheat grains were found to be more sensitive to low-temperature than major minerals (P, K, and Ca). In general, low temperatures occurring in the early growth stage of wheat have significant and persistent effects on the enrichment level of mineral components in grains at final maturity. This study included a comprehensive evaluation of the mineral nutritional quality of grains under different low-temperature treatments, and the results can provide beneficial information for the bio-fortification of crops under future climate scenarios and help alleviate the intensified ‘hidden hunger’ trend. However, constrained by the resources and workload, this study only focused on the impact of pre-anthesis low temperature on grain mineral components at maturity. Further exploration is needed to elucidate the effects of low temperature on the processes of mineral absorption and transport in wheat in order to inform the breeding of wheat with higher cold tolerance. Moreover, the response of minerals to low temperature varies among different cultivars. Thus, the results of this study need to be further validated with more cultivars.

## Data availability statement

The original contributions presented in the study are included in the article/**Supplementary Material**. Further inquiries can be directed to the corresponding author.

## Author contributions

Conceptualization: LL, XQ, and WC. Methodology: XH and BL. Data curation: WJ, XH, and MK. Writing—original draft preparation: WJ and LL. Writing—review and editing: LL and LT. Funding acquisition: YZ, LL, and BL. All authors contributed to the article and approved the submitted version.
