# Supplementary material for: Effects of pre-anthesis low-temperature stress on the mineral components in wheat grains
Source: Front Plant Sci. 2023 Jul 27;14:1221466. doi: 10.3389/fpls.2023.1221466 (PMC10413566; doi:10.3389/fpls.2023.1221466)
Supplement: Supplementary file 1 [file Image_1.pdf]

Supplementary Material

Effects of pre-anthesis low-temperature stress on the mineral components in wheat grains

Wenbin Ji *, Xinyi Hu, Meng Kang

*** Correspondence:** Leilei Liu: liuleilei@njau.edu.cn

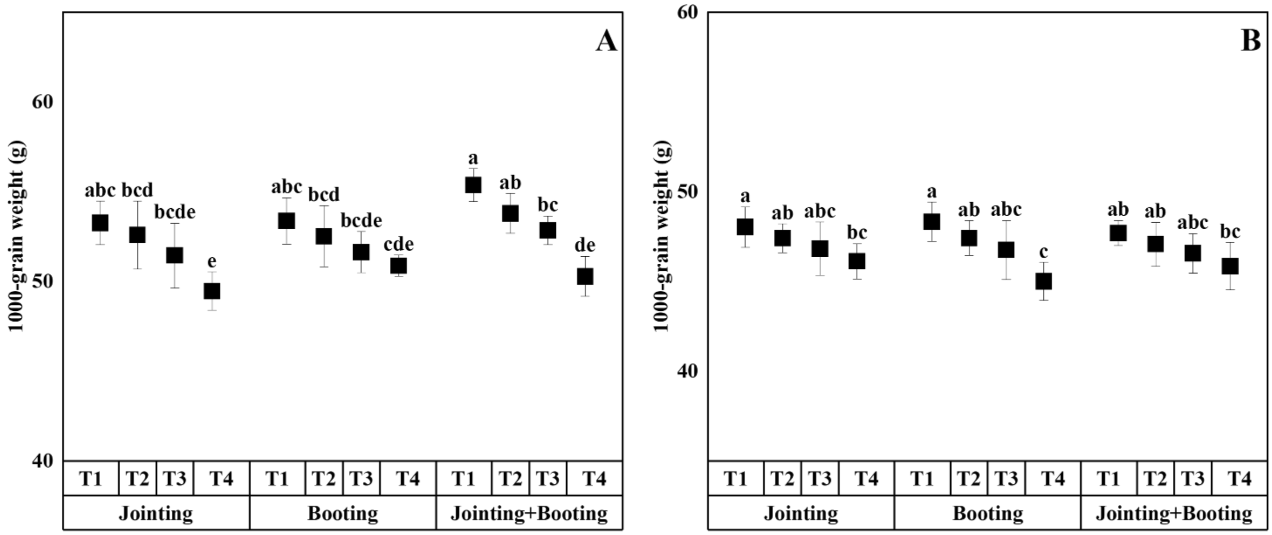

Supplementary Figure 1. The 1000-grain weight under different low-temperature treatments in Yangmai16 (A) and Xumai30 (B). Vertical bars represent the standard deviation of the mean. Different lowercase letters indicate significant differences at $p < 0.05$.
